# Nanoelectronic primary thermometry below 4 mK

**DOI:** 10.1038/ncomms10455

**Published:** 2016-01-27

**Authors:** D. I. Bradley, R. E. George, D. Gunnarsson, R. P. Haley, H. Heikkinen, Yu. A. Pashkin, J. Penttilä, J. R. Prance, M. Prunnila, L. Roschier, M. Sarsby

**Affiliations:** 1 Department of Physics, Lancaster University, Bailrigg, Lancaster LA1 4YB, UK; 2 VTT Technical Research Centre of Finland, P.O. Box 1000, 02044 VTT Espoo, Finland; 3 Lebedev Physical Institute, Moscow 119991, Russia; 4 Aivon Oy, Valimotie 13A, 00380 Helsinki, Finland

## Abstract

Cooling nanoelectronic structures to millikelvin temperatures presents extreme challenges in maintaining thermal contact between the electrons in the device and an external cold bath. It is typically found that when nanoscale devices are cooled to ∼10 mK the electrons are significantly overheated. Here we report the cooling of electrons in nanoelectronic Coulomb blockade thermometers below 4 mK. The low operating temperature is attributed to an optimized design that incorporates cooling fins with a high electron–phonon coupling and on-chip electronic filters, combined with low-noise electronic measurements. By immersing a Coulomb blockade thermometer in the ^3^He/^4^He refrigerant of a dilution refrigerator, we measure a lowest electron temperature of 3.7 mK and a trend to a saturated electron temperature approaching 3 mK. This work demonstrates how nanoelectronic samples can be cooled further into the low-millikelvin range.

## Abstract (web summary)

When cooled to the millikelvin scale, nanoelectronic structures can become thermally detached from their environment, limiting nanoscale electronic thermometry. Here, the authors demonstrate the robust cooling of optimally-designed Coulomb blockade thermometer devices down to the millikelvin scale.

## Graphical abstract

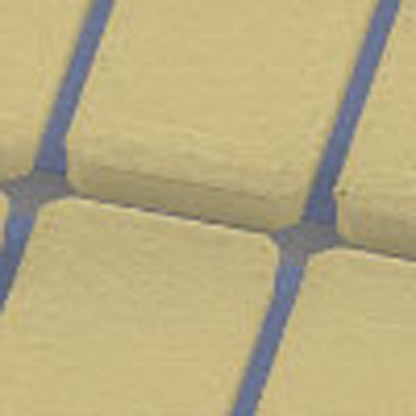

Understanding how to obtain and measure electron temperatures approaching 1 mK has the potential to open a new regime for studying nanoelectronics and pave the way towards pioneering sub-millikelvin techniques^1^. This would benefit numerous areas of activity; for example, investigations of the fractional quantum Hall effect in two-dimensional electron gases^2^,^3^ and solid-state quantum technologies including superconducting and semiconducting qubits^4^,^5^. To access these temperatures one must minimize parasitic heating and internal Joule heating, and maximize the coupling to cold contact wires and phonons in the host lattice, all the while overcoming the decrease in electron–phonon coupling and electrical heat conduction as temperatures drop^6^.

Here we study Coulomb blockade thermometers (CBTs) that have been designed to operate significantly below 10 mK and demonstrate cooling of electrons in a nanoelectronic device to below 4 mK. A CBT consists of an array of Coulomb-blockaded metallic islands connected by tunnel junctions. The conductance of the array is temperature dependent, due to the balance between thermal excitations and an electrostatic barrier to single electron tunnelling across the islands^7^,^8^. CBTs typically function over a decade of temperature and have previously been demonstrated to work at temperatures as low as 7.5 mK (refs 9, 10). Perhaps most importantly, they can be viewed as a primary thermometer of their internal electron temperature. We have used CBTs as a diagnostic tool to quantify and optimize the thermal environment of a nanoelectronic device and to unambiguously determine the local electron temperature. In the future, similar CBTs could be used to probe the electron temperature of nearby samples with thermal contact provided by direct electrical connections. Furthermore, the techniques that are used to cool the CBTs can be used to cool other nanoelectronic samples.

## Results

### Structure of the thermometer devices

**Figure 1:**
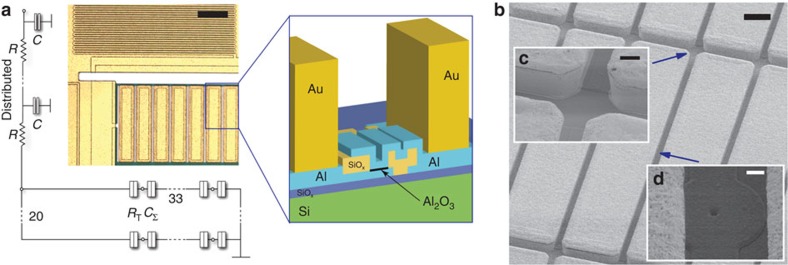
Details of the CBT device structure. (**a**) Optical micrograph of the CBT with equivalent circuit diagram and schematic cross-section of the structure. Scale bar on the optical micrograph, 10 μm. The CBT is formed of 32 × 20 metallic islands of capacitance *C*_Σ_ connected in an array by tunnel junctions of resistance *R*_T_, as shown in the circuit diagram. Connection to the array is made via on-chip *RC* filters comprising a meandering electrode sandwiched between large-area grounded metal films, separated by 250 nm SiO_*x*_. Each filter has a distributed resistance *R*≈500 Ω and capacitance *C*≈10 pF. The schematic cross-section shows one Al_2_O_3_ tunnel junction connecting two Al islands, with Au thermalization blocks on top of each. (**b**) Scanning electron microscopic (SEM) image showing Au thermalization blocks. Scale bar, 20 μm. (**c**) The sidewalls of four adjacent Au blocks. Scale bar, 4 μm. (**d**) One tunnel junction connecting two adjacent blocks. Scale bar, 1 μm.

The structure of the CBTs studied here is shown in Fig. 1. Devices are fabricated using an *ex situ* tunnel junction process^11^, which provides excellent tunnel junction uniformity^12^, and has also been used to fabricate superconducting qubits^13^ (see Methods for details). Efficient thermal coupling between electrons and phonons in the metallic islands of the CBT is critical for reaching low electron temperatures^14^,^15^. The electron–phonon heat flow *P*_ep_ is described by the material-dependent electron–phonon coupling constant Σ and the volume of the metallic island Ω,


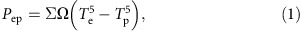


where *T*_e_ is the electron temperature and *T*_p_ is the phonon temperature^16^. To minimize *T*_e_, the island volume should be large and the material chosen to maximize Σ. We use electroplated Au on top of the CBT islands to increase their volume to nominal 5 × 205 × 38.5 μm^3^ (see Methods for details). The effective electron–phonon coupling in these islands, with a relatively large volume and a high coupling constant^17^ in Au Σ=2.4 × 10^9^ WK^−5^ m^−3^, is estimated to be more than two orders of magnitude larger than in previous CBTs fabricated using the *ex situ* junction process^18^.

In addition to efficient thermalization of the CBT itself, it is important to cool the incoming leads through robust thermal anchoring and heavy electromagnetic filtering^19^. We improve the chain of thermalization and filtering by including on-chip resistive meander structures in line with all electrical contacts. These form a distributed resistive-capacitive chain with a cutoff frequency ≈40 MHz. Similar filters based on a large area capacitor and tunnel junctions in series have previously been incorporated in a CBT^18^.

### CBT characteristics above 7 mK

**Figure 2:**
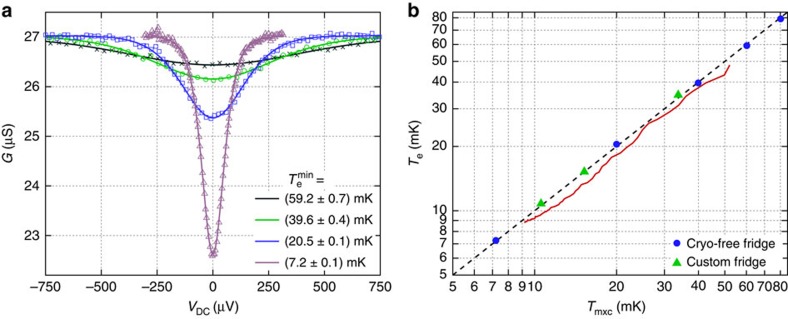
CBT behaviour between 80 and 7 mK in two dilution refrigerators. (**a**) CBT conductance *G* versus measured bias voltage *V*_DC_ at four temperatures. Symbols show measured values and lines show best fits to the calculated ideal conductance. The warmest measurements (crosses, circles and squares) are fitted simultaneously to calibrate the sensor, giving *C*_Σ_=236.6 fF and *R*_T_=22.42 kΩ. The coldest measurement (triangles) is fitted using this calibration. The minimum electron temperatures 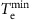 are in close agreement with the refrigerator temperature measured by a RuO_2_ thermometer: 59.9, 40.1, 20.0 and 7.2 mK respectively. The uncertainties on 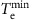 are calculated from uncertainties in the fitted parameters. (**b**) CBT electron temperature *T*_e_ versus refrigerator temperature *T*_mxc_. Symbols show 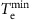 from fits to conductance dips measured in the cryogen-free refrigerator (circles) and the custom refrigerator (triangles). Error bars are within the symbols. The solid curve shows *T*_e_ determined by monitoring the conductance *G*_0_ (at *V*_DC_=0) as the cryo-free fridge cools over 35 min from 52 to 9 mK, showing that the CBT has a stronger thermal link to the refrigerator than the RuO_2_ thermometer, leading to the thermal lag (*T*_mxc_≥*T*_e_) during this time. The dashed line shows *T*_e_=*T*_mxc_.

Figure 2 shows the behaviour of a CBT fabricated using the process described above, focusing on temperatures between 7 and 80 mK. The sensor is measured in both a commercial cryogen-free dilution refrigerator (Bluefors Cryogenics LD250) with a base temperature ≈7 mK and in a custom dilution refrigerator manufactured at Lancaster University^20^ with a base temperature ≈2.5 mK. The conductance of the CBT is measured in a current-driven four-wire configuration, with the drive current and voltage amplification provided by an Aivon PA10 amplifier. A small AC excitation (typically 5 pA≤*I*_AC_≤100 pA) is added to the DC bias *I*_DC_, allowing the differential conductance *G* to be measured with a lock-in amplifier.

In both refrigerators, the CBT is in a vacuum and housed in a gold-plated copper package (Aivon SH-1) that is attached to the mixing chamber plate. The package includes RC filters with a cutoff frequency ≈300 kHz. Electrical contacts to the CBT are thermalized in additional cold *RC* filters potted in Eccosorb CR 124 (Aivon Therma), which are also attached to the mixing chamber plate. The filters have a cutoff frequency ≈15 kHz.

As shown in Fig. 2a, the CBT conductance dips around zero bias and the dip becomes deeper and narrower at lower temperatures. Its full-width at half maximum is related to temperature by *V*_1/2_≈5.439*Nk*_B_*T*/*e*, where *N* is the number of tunnel junctions in series^7^. This result does not account for self-heating in the sensor and so is only applicable when *T*_e_=*T*_p_≡*T*. A more practical parameter to determine temperature is the zero-bias conductance *G*_0_, which has an approximate analytic relation to temperature^21^


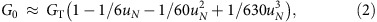


where *u*_*N*_≡*E*_*C*_/*k*_B_*T* is the dimensionless inverse temperature, *E*_*C*_≡[(*N*−1)/*N*]*e*^2^/*C*_Σ_ is the charging energy of the system, *C*_Σ_ is the total capacitance of each island and *G*_T_ is the asymptotic conductance. When *u*_*N*_<2.5, the temperature measurement error is <2.5% (ref. 15). Thus, if *C*_Σ_ and *G*_T_ are known, it is possible to determine *T* by measuring only *G*_0_. The most complete method to determine temperature is using a full tunnelling model to calculate *G*(*V*_DC_) numerically^7^. We use this last approach to find *C*_Σ_ and *G*_T_ for the device, and these parameters are then used to find the temperature from subsequent measurements.

Numerical calculations of conductance are made using an algorithm derived from the free, open-source library pyCBT (see Methods for further details). In addition, we account for overheating in the sensor by predicting the electron temperature *T*_e_ in the islands using a model for the heat flow *P* into each island,


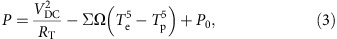


where the first term is Joule heating at tunnel junctions of resistance *R*_T_, the second term is heat flow to phonons and *P*_0_ accounts for parasitic heating. For a given phonon temperature, the minimum electron temperature 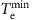 is found at *V*_DC_=0. If *P*_0_ is small, then 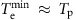.

Figure 2a shows the result of fitting the calculated *G*(*V*_DC_) simultaneously to three measurements made between 20 and 60 mK. The fit parameters are *R*_T_, *C*_Σ_ and *T*_P_ for each measurement. The fit is found to be insensitive to the value of *P*_0_ and hence parasitic heating is assumed to be negligible at these temperatures. Having calibrated the CBT, the fitted *C*_Σ_ is used to fit further measurements with *T*_p_ and *R*_T_ as the free parameters. An example is given in Fig. 2a, where the electron temperature is found to be 7.2±0.1 mK at a refrigerator temperature of 7.2 mK. Figure 2b shows that the electron temperature measured by the CBT is in close agreement with the refrigerator temperature *T*_mxc_ in both refrigerators between 7 and 80 mK. Details of how *T*_mxc_ was measured in each fridge can be found in the Methods section.

### CBT characteristics below 7 mK

**Figure 3:**
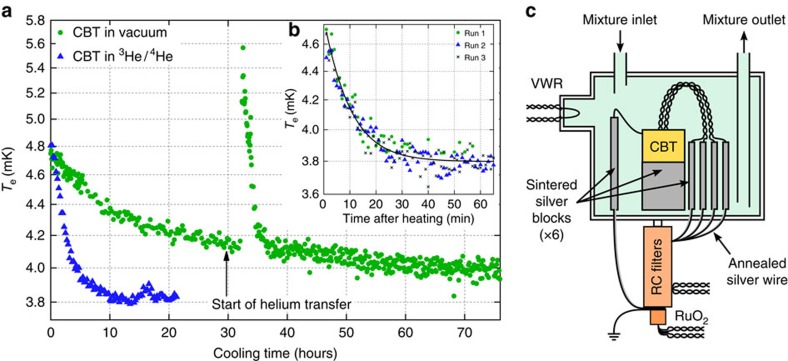
Thermalization of two CBTs at a refrigerator temperature ≤2.8 mK. (**a**) Cooling of one CBT in vacuum (circles) and one immersed in the ^3^He/^4^He refrigerant of the dilution refrigerator (triangles). In both cases, the CBTs are cooling after being warmed above 10 mK by temporarily increasing the refrigerant temperature. The CBT in vacuum is extremely slow to thermalize. By comparison, the CBT immersed in ^3^He/^4^He thermalizes significantly faster. (**b**) Cooling of the immersed CBT after it has been heated by a large DC drive current (50, 40 and 30 nA for run 1, 2 and 3, respectively). Fitting to an exponential decay (solid line) yields a time constant of 570 s and a saturation temperature of 3.8 mK. (**c**) Schematic of the immersion cell used to cool a CBT in the ^3^He/^4^He mixture of a dilution refrigerator.

We investigate the behaviour of the CBT below 7 mK in the custom dilution refrigerator. Figure 3a shows the electron temperature of the sensor when in vacuum gradually cooling below 4 mK, while the refrigerator was held at *T*_mxc_≲2.8 mK. Here, *T*_e_ is determined from the value of *G*_0_, which is found by measuring *G* over a small range of *V*_DC_ (≈30 μV) close to *V*_DC_=0. We observe an extremely long equilibration time (over 3 days) but a rapid cooling of the CBT following a heating event (a refill of the liquid helium bath that briefly heats the CBT above 5.5 mK and the fridge above 3.5 mK). This suggests that the thermal contact between the CBT and the refrigerator is relatively strong, and that its slow cooling is probably due to heat leak from an external warm object.

A second CBT was immersed in the ^3^He/^4^He refrigerant of the custom dilution refrigerator to improve thermal coupling and better isolate external sources of heating. A schematic of the immersion cell is shown in the Fig. 3c. Sintered silver blocks increase the thermal contact between the refrigerant and the incoming contact wires^2^. Several sinters are attached to the sensor package, to each of the four measurement wires and to a grounding wire for the package and the *RC* filters. The immersed CBT is found to equilibrate much faster, as shown in Fig. 3a, reaching *T*_e_≈3.8 mK at *T*_mxc_≈2.7 mK. It is important to note that even when *T*_e_ is elevated above *T*_p_ and *T*_mxc_, the CBT remains a primary thermometer of its internal electron temperature.

To study the CBT at 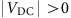, the time needed for the sensor to reach thermal equilibrium after a change of Joule heating needs to be known. Figure 3b shows the relaxation of *T*_e_ after the CBT has been heated by a large drive current for long enough to reach thermal equilibrium (>30 min). The subsequent value of *T*_e_ is measured by scanning close to *V*_DC_=0, where Joule heating should be negligible. The relaxation of *T*_e_ is found to have a time constant of 570 s.

**Figure 4:**
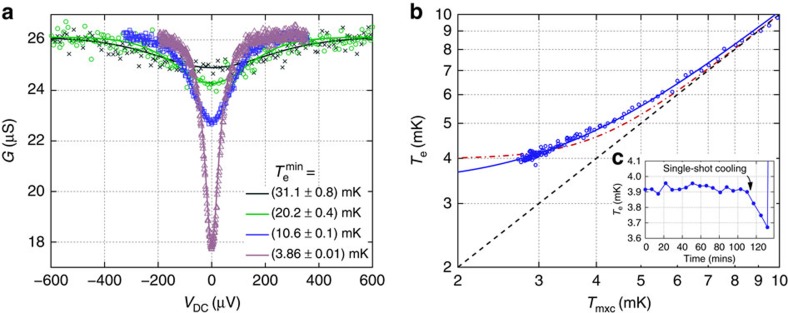
Characteristics of a CBT immersed in ^3^He/^4^He refrigerant. (**a**) Fitting to the warmest three measurements gives *C*_Σ_=209.5 fF and *R*_T_=23.21 kΩ. The fitted minimum electron temperatures 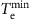 for the warmest three curves are in reasonable agreement with the refrigerator temperatures as measured by the vibrating wire resonator (VWR) thermometer: 29.4, 19.0 and 10.5 mK, respectively. (**b**) Measured electron temperature in the CBT as the refrigerator cooled steadily from 10 to 2.7 mK over a period of 12 h. The solid curve shows a fit of the form 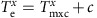, yielding an exponent *x*=2.7. The dot-dashed curve shows a best fit of 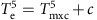. The dashed line shows *T*_e_=*T*_mxc_. (**c**) The measured *T*_e_ as the refrigerator was temporarily cooled in single-shot mode to 2.2 mK, reaching a lowest *T*_e_ below 3.7 mK.

Figure 4a shows the calibration of the immersed sensor. The three warmest measurements are fitted simultaneously to determine *C*_Σ_=209.5 fF and *R*_T_=23.21 kΩ. The fitted temperatures agree with *T*_mxc_ to within 6%. Given the agreement between the fitted 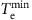 and *T*_mxc_, we can assume that parasitic heating is still negligible down to 10 mK.

The coldest measurement in Fig. 4a is fitted using the above values, yielding a minimum electron temperature of 3.86±0.01 mK. This measurement was made over a period of 7 h, to ensure that the CBT was in thermal equilibrium at each value of *V*_DC_. At these temperatures, the parasitic heating of the CBT is now significant and 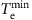 does not match the refrigerator temperature of *T*_mxc_=2.7 mK. To fit this conductance dip, the thermal model, equation 3, is used with *T*_p_=*T*_mxc_, and with the parasitic heating *P*_0_ and the electron–phonon coupling ΣΩ as free parameters.

Figure 4b shows how the CBT electron temperature diverges from the refrigerator temperature below ≈7 mK. Here the value of *T*_e_ is found by measuring *G*_0_ close to *V*_DC_=0 and so Joule heating can be neglected. The lowest temperature reported by the CBT is below 3.7 mK when operating the fridge in single-shot mode (see Fig. 4c).

## Discussion

The overheating of the sensor at *V*_DC_=0 constrains the value of *P*_0_(ΣΩ)^−1^ in the fit to the coldest measurement in Fig. 4a. However, the parasitic heating is not large enough to reliably separate the values of *P*_0_ and ΣΩ in the fitting. Qualitatively, the fits suggest that *P*_0_≥300aW per island and ΣΩ is at least four times larger than expected from the nominal size of the thermalization blocks and the literature value of Σ for Au^17^. It is not possible to determine an upper bound on *P*_0_ without constraining ΣΩ. It is worth noting that the power required to measure the CBT conductance (∼1aW per island due to Joule heating from *I*_AC_) is much lower than our estimate of *P*_0_. As such, we believe that CBTs of this type can be operated at still lower temperatures by further reducing the parasitic heating.

The functional form of *T*_e_ versus *T*_mxc_, as shown in Fig. 4b, should have the same temperature dependence as the dominant thermalization mechanism, that is, *T*^5^ for electron–phonon coupling. However, other power laws have been observed^9^. Here we find that the best fit of 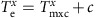 gives *x*=2.7 and a saturated *T*_e_ of *c*^1/*x*^=3.4 mK. The fitted exponent *x* cannot be confirmed by fits to the conductance dips in Fig. 4a, because the overheating is still relatively weak, even at the lowest temperatures, and there is little effect on the shape of the dip. We find that a thermal model with a *T*^3^ thermalization term fits the conductance dips equally well as a model using *T*^5^.

The saturation of the measured CBT temperature below 7 mK could be caused by parasitic heating of the islands or excess voltage noise across the tunnel junctions. It is possible that the operating temperature could be lowered by reducing parasitic heating through better shielding and by lowering the voltage noise in the measurement circuit. To understand the cause of saturation in more detail, or to test an improved measurement environment, this sensor would need to be cooled closer to 1 mK.

In conclusion, the CBTs described here have been shown to operate as reliable primary thermometers of electron temperature down to 3.7 mK. The large thermalization blocks incorporated in the device and a relatively low level of parasitic heating ensure that the electron subsystem in the sensor is well coupled to the phonon subsystem down to ≈7 mK. An immersion cell is shown to improve thermal coupling between a CBT and a dilution refrigerator. This allows the onset of overheating to be observed below 7 mK, and although the presence of overheating can be seen, the effect is sufficiently weak that the sensor will need to be cooled further to fully characterize the thermalization mechanisms.

## Methods

### Device fabrication

The CBT devices are fabricated using an *ex situ* tunnel junction process^11^. The Al films that define the CBT circuit have a thickness of 250 nm. Tunnel junctions between sections of Al are formed by an insulating layer of 250 nm SiO_x_ deposited by plasma-enhanced chemical vapour deposition (PECVD). The junctions have a nominal diameter 0.8 μm and a resistivity ∼10 kΩ μm^2^. The substrate is undoped Si with 300 nm thermal oxide on the surface.

The island thickness achievable with the *ex situ* tunnel junction process or other deposition techniques used for tunnel junction devices is typically up to 1 μm. Thicker films suffer from stress build-up, causing poor adhesion between the film and the substrate. This is a severe problem at mK temperatures where poor adhesion can lead to poor thermalization and even mechanical failure due to thermal motion during cool down. We avoid these problems by using a combination of the *ex situ* process followed by masked electroplating of Au on top of the CBT islands^22^, which we refer to as thermalization blocks. Electroplating can produce ∼10 μm-thick, low-stress films and here we choose a nominal thickness of 5 μm for the thermalization blocks.

### Refrigerator thermometry

Two different dilution refrigerators are used in the experiments described above. In the commercial refrigerator, the mixing chamber temperature *T*_mxc_ is measured by a calibrated RuO_2_ resistor (Sensor model RU-1000-BF0.007 supplied and calibrated by Bluefors Cryogenics) in contact with the mixing chamber plate. In the custom refrigerator, *T*_mxc_ is measured using a conventional vibrating wire resonator viscometer immersed in the saturated dilute phase of the ^3^He/^4^He refrigerant in the mixing chamber^23^,^24^. The vibrating wire resonator is validated by comparison with a calibrated RuO_2_ resistor (calibrated to 20 mK and supplied by Lake Shore Cryogenics). This resistor is thermally connected to the refrigerant via an immersed pad of sintered silver.

### Data and software availability

All data used in this paper are available at http://dx.doi.org/10.17635/lancaster/researchdata/31, including descriptions of the data sets. The python-based pyCBT software library is freely available from Aivon Oy at https://github.com/AivonOy/pyCBT.

## Additional information

**How to cite this article:** Bradley, D. I. *et al.* Nanoelectronic primary thermometry below 4 mK. *Nat. Commun.* 7:10455 doi: 10.1038/ncomms10455 (2016).
