# Erratum: Nanoelectronic primary thermometry below 4 mK

**DOI:** 10.1038/ncomms11678

**Published:** 2016-06-03

**Authors:** D. I. Bradley, R. E. George, D. Gunnarsson, R. P. Haley, H. Heikkinen, Yu A. Pashkin, J. Penttilä, J. R. Prance, M. Prunnila, L. Roschier, M. Sarsby

Nature Communications 7: Article number: 10455 10.1038/ncomms10455 (2016); Published: 01272016; Updated: 06032016

The affiliation details for L. Roschier are incorrect in this Article. The correct affiliation details for this author are given below:

Aivon Oy, Valimotie 13A, 00380 Helsinki, Finland.

